# Retraction: Diet and Lifestyle Impact on Rheumatoid Arthritis: A Comprehensive Review

**DOI:** 10.7759/cureus.r146

**Published:** 2024-07-19

**Authors:** Kartikey V Shekhar, Mrunmayee M Pathak, Gajanan Pisulkar

**Affiliations:** 1 Medical Education, Jawaharlal Nehru Medical College, Datta Meghe Institute of Higher Education and Research, Wardha, IND; 2 Orthopaedics, Jawaharlal Nehru Medical College, Datta Meghe Institute of Higher Education and Research, Wardha, IND

This article has been retracted by the Editor-in-Chief due to severe errors in the selection of references. References 15-19 do not match the corresponding citations found in the article. These errors call into question the validity of the review as a whole, and as a result, it has been determined that this article must be retracted in order to correct the scientific record.

